# Case Report: Relevance of an Accurate Diagnosis and Monitoring of Infective Dermatitis Associated With Human T-Lymphotropic Virus Type 1 in Childhood

**DOI:** 10.3389/fmed.2021.758352

**Published:** 2021-11-10

**Authors:** Paula Benencio, Nicolás Ducasa, Lourdes Arruvito, Inés Irurzun, Laura Praino, Magdalena Lamberti, María Beraza, Carolina Berini, Mirna Biglione

**Affiliations:** ^1^Instituto de Investigaciones Biomédicas en Retrovirus y SIDA (INBIRS), CONICET- Universidad de Buenos Aires, Buenos Aires, Argentina; ^2^Unidad de Dermatología, Hospital de Niños Dr. Ricardo Gutiérrez, Buenos Aires, Argentina; ^3^Unidad de Infectología, Hospital de Niños Dr. Ricardo Gutiérrez, Buenos Aires, Argentina

**Keywords:** infective dermatitis, HTLV-1, pediatric, antibiotic, Argentina, case report

## Abstract

Human T-lymphotropic virus type 1 (HTLV-1) is a neglected retrovirus distributed worldwide and the ethiological agent of several pathologies, such as adult T-cell leukemia/lymphoma (ATLL), a chronic myelopathy known as HTLV-1 associated myelopathy/tropical spastic paraparesis (HAM/TSP) and infective dermatitis associated with HTLV-1 (IDH). HTLV-1 presents tropism for CD4^+^ T cells and induces deregulation of the cytokine profile. IDH is a severe, chronic superinfected eczema generally associated with *Staphylococcus aureus* and/or *Streptococcus beta haemolyticus* infection that responds partially to antibiotic therapy but prompt recurrence develops upon treatment withdrawal. IDH could be a risk factor for progression toward both HAM/TSP and ATLL and, similarly to other diseases associated with HTLV-1, it is sub-diagnosed particularly in non-endemic areas. Here, we present a case of IDH in a young boy living in Buenos Aires with symptoms since 2010, at the age of 5. HTLV-1 infection was suspected and confirmed in 2016. The patient exhibited chronic dermatosis with exudative eruption involving mainly the scalp, retroauricular regions, neck and abdomen. Clinical evaluations, routine laboratory tests, full blood count, and HTLV-1 diagnosis for this case are included.

## Introduction

Human T-lymphotropic virus type 1 (HTLV-1) is a neglected retrovirus distributed worldwide in endemic regions such as Japan, Rumania, Iran, Jamaica, Western Africa and South America, and mostly diagnosed elsewhere in immigrants from endemic areas. HTLV-1 causes adult T-cell leukemia/lymphoma (ATLL) and a chronic demyelinating neurologic disease known as HTLV-1 associated myelopathy/tropical spastic paraparesis (HAM/TSP), but it is also associated with several inflammatory disorders like HTLV-1 related uveitis, chronic respiratory diseases, strongyloidiasis, and infective dermatitis associated with HTLV-1 (IDH) (1). HTLV-1 presents tropism for CD4^+^ T cells, particularly the CD4^+^ CD25^+^ CCR4^+^ subset, and produces deregulation of the cytokine profile (2). IDH was first described in children in Jamaica, where an association between HTLV-1 infection and a distinct pattern of dermatitis was recognized in 1990 (3). IDH is a severe, chronic superinfected eczema generally associated with *Staphylococcus aureus* and/or *Streptococcus beta haemolyticus* infection. Diagnosis is based on a modified version of La Grenade's criteria, published in 2012 by de Oliveira et al. (4) which define indispensable criterias such as (I) presence of erythematous-scaly, exudative, and crusted lesions involving at least three areas, including the scalp and retroauricular regions; (II) recurring nature of the lesions; and (III) confirmed HTLV-1 infection (Table 1). IDH onset is usually observed in early childhood and disappears during adolescence at a mean age of 15 years although it has also been described to persist until 26 years of age; and it can also develop in adulthood (5, 6). The treatment of IDH is based on the chronic administration of antibiotics that act against *Staphylococcus* and *Streptococcus*. Good results have been obtained with the use of trimethoprim/sulfamethoxazole (T/S). Generally, IDH responds to antibiotic therapy but prompt recurrence develops upon treatment withdrawal. IDH is associated with high HTLV-1 proviral loads (PVL), and characterized by a predominantly Th1 immune response (specialized in response to intracellular infections, with secretion of IL-2, and IFNg, and expression of *T*-bet transcription factor), with CD8^+^ T cell infiltrates in skin. CD4^+^/CD8^+^ T cell ratio is usually elevated. Tumor necrosis factor alpha (TNF-α) and interferon gamma (IFN-ɤ) levels are also frequently increased in IDH patients when compared to asymptomatics (7). IDH is associated with an increased risk of HAM/TSP and ~30% of children with IDH were reported to develop this neurological disease in Latin America (7, 8). An endemic focus for HTLV-1 can be found in Northwestern Argentina but IDH has not been previously documented in children in the country (9). Here, we report a case of IDH in a 12-year-old child living in Buenos Aires, Argentina.

**Table 1:** La Grenade's criteria modified by de Oliveira et al. (4).

**IDH diagnostic criteria**	
1.	Presence of erythematous-scaly, exudative, and crusted lesions of the scalp, retroauricular areas, neck, axillae, groin, paranasal and perioral skin, ears, thorax, abdomen, and other sites.
2.	Crusting of nostrils.
3.	Chronic relapsing dermatitis with prompt response to appropriate therapy but prompt recurrence on discontinuation of antibiotics.
4.	Diagnosis of HTLV-1 infection (by serological or molecular biological testing).

*From the four statements of this criteria, 1, 3, and 4 are mandatory. To fulfill criteria 1, involvement of at least three of the sites is required, including involvement of the scalp and retroauricular areas*.

## Case Presentation

**Figure 1:**
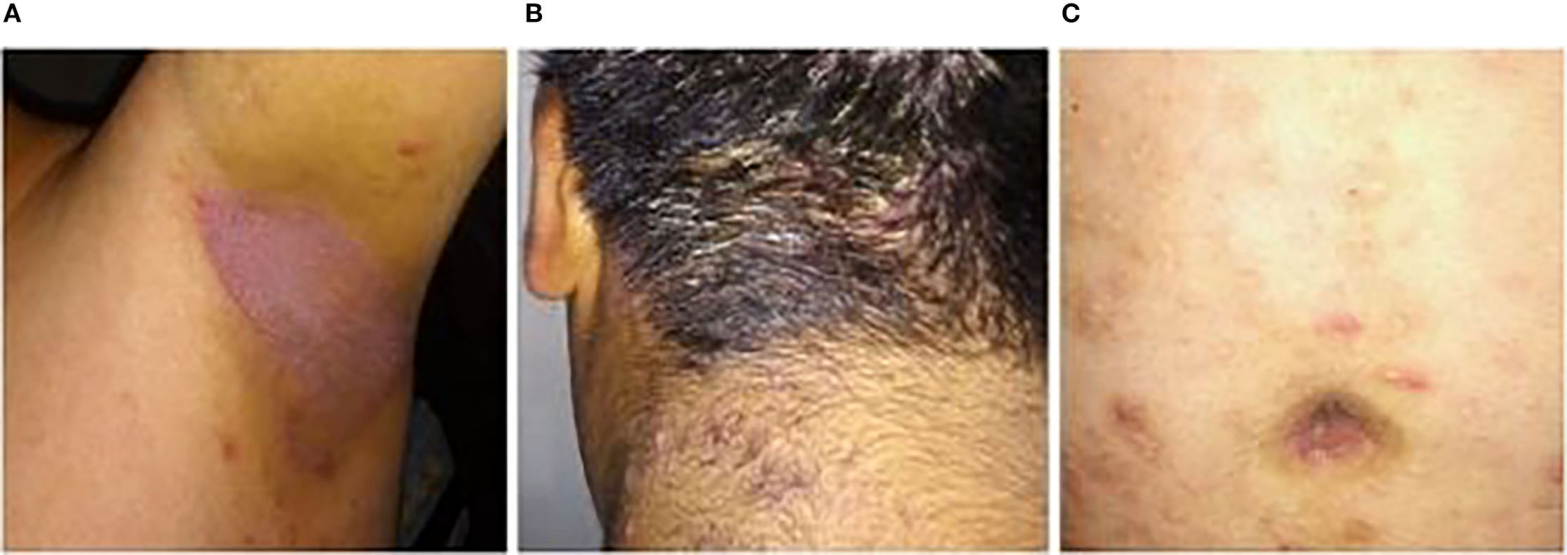
Erythematous and scaly plaque in axillae **(A)**, chronic exudative eczema in scalp **(B)**, and inflammatory papules involving the abdomen **(C)** in a 12-year-old child with infective dermatitis associated with HTLV-1.

In 2010, a 5-year-old child attended a Public Hospital in Buenos Aires for the first time, with a history of relapsing atopic dermatitis and urticaria. The patient had been breastfed until the age of five. His family was of lower middle socioeconomic status, with no remarkable clinical history. He was of Amerindian descent from his father's side. On physical examination, he was mildly overweight with +1 <z ≤ +2, and presented an exudative eczema in axillae, neck, groins, inframammary folds, and periumbilical area. Retroauricular regions and auricles were also compromised, and the patient complained of ear recurrent discharge. Eczema was prominent on the scalp, showing thick scaling and chronic exudation. There were scabs on nostrils and he suffered from nasal discharge. There were also inflammatory papules on the neck, abdomen and back (Figure 1). Pruritus was mild. Overall, the patient looked healthy.

Seborrheic dermatitis and tinea capitis were suspected and ruled out. Normal values were observed in blood count, kidney and liver function, electrolytes, and urine analysis. Laboratory tests showed high IgE levels (>1,000 IU) and dyslipidemia. Parasitological analysis was negative. The patient was treated with topical corticosteroids and topical calcineurin inhibitors for skin lesions; topical salicylic acid, coal tar, and betamethasone lotions for the scalp. He also received antihistamines and antibiotics, such as cephalosporins, T/S. On antibiotic courses he experienced a good response, but relapsed after discontinuation.

At the age of seven, he was hospitalized due to exacerbation of his symptoms, skin infection and blepharoconjunctivitis which improved with empiric antibiotics. Blood analysis was within normal values and both blood and urine cultures were negative for fungi and bacteria. Inverted psoriasis was suspected. Skin biopsy revealed psoriasiform dermatitis with epidermal hyperplasia, parakeratosis with few polymorphonuclear cells, acanthosis and hypergranulosis, elongations of vascular connective axes, edematous dermis with mononuclear inflammatory infiltrates and isolated melanophages. At the age of eight, due to the persistence of his psoriasiform-like dermatitis, narrow-band ultraviolet B (NB-UVB) therapy was initiated, and subsequently methotrexate. There was no favorable response to any of them so treatment was discontinued. Skin affection impacted not only on the child's school attendance but also on social interactions. The child's embarrassment and some discrimination episodes prevented him from participating in physical activities and social meetings, and he didn't like to be photographed.

After these events, at the age of 11 in 2016, he was re-evaluated at the dermatology unit. Infective dermatitis was suspected. A new skin biopsy revealed once again spongiosis and acanthosis, but with a perivascular lymphocytic infiltrate. Phenotypical analysis was performed by flow cytometry, which showed a decreased ratio of naive (49.6%) vs. memory effector (20.6%) CD8^+^ lymphocyte population, compatible with viral infection. Thus, HTLV-1 infection was suspected and confirmed at INBIRS Institute in Buenos Aires. Specific antibodies anti-HTLV-1 were detected by enzyme-linked immunoassay (ELISA) (ELISA HTLV I&II Ab, ULTRA version, Diapro), and infection was confirmed by nested polymerase chain reaction (n-PCR, in-house) after DNA extraction (ADN PuriPrep-S kit, Highway, Inbio). PVL was determined to be 11.5 copies/100 peripheral blood mononuclear cells (PBMC) by quantitative PCR (qPCR) (SybrGreen, ThermoFisher, MA, USA). His mother was also found positive for HTLV-1, but none of his siblings. Human immunodeficiency virus (HIV) and other immunodeficiencies were excluded. IDH was confirmed based on La Grenade's modified criteria (4). Blood tests were normal, ruling out *T*-cell leukemia/lymphoma. Stool parasitological and *Strongyloides stercoralis* tests were negative. Neurological evaluation and brain magnetic resonance imaging (MRI) were normal. Nonetheless, IgE levels increased (3,314 IU). Cytokines in plasma were also analyzed. An increase in interleukin (IL)-6 and IL-9 concentrations was determined while no increase in plasma levels of Th1 (IFNg, IL-2), Th2 (IL-2, -4, -5, -13) or Th17 (IL-17A, -17F, -22) cytokine profiles was observed by LEGENDplex immunoassay (BioLegend). Specific allergen radio allergo sorbent test (RAST) was requested, with a positive result for Dermatophagoides. A specific immunotherapy was indicated with slight improvement.

Once diagnosed, appropriate chronic low dose treatment (alternating antibiotics against gram positive cocci) was indicated to avoid bacterial superinfection of the skin. T/S, minocycline and clindamycin were used according to local resistance patterns. On chronic antibiotic therapy, no further hospital admissions were required. Prophylaxis with T/S was started and the patient experienced partial remission although relapses of folliculitis and erythematous scaly crusty lesions were present. For acute skin infections, 10-day courses of these drugs or clindamycin were alternated. *Staphylococcus aureus* decontamination was periodically indicated with mupirocin 2% ointment and chlorhexidine. The clinical status evolved with recurrences but favorably. The patient continued to present lower intensity exudative and crusty eczema in folds, scalp, and retroauricular regions; generalized papular eruption involving the face and trunk.

At the age of 14, in 2019, a complete urine test showed glomerular hematuria (>50% dysmorphic red blood cells) and significant proteinuria. Blood pressure was always within normal limits. He was then followed up in the nephrology department. A significant increase in the PVL values (22 copies/100 PBMC) was observed when compared to the first measurement in 2016.

At the age of 15 (in 2020), prophylaxis with minocycline was prescribed, during which period less soft tissue infections were observed. However, the patient still suffered from relapsing chronic dermatitis, presenting recurrences when discontinuing or lowering the antibiotics dose, especially in hot and humid weather. Minocycline was interrupted 6 months later because of a photosensitivity reaction.

**Figure 2:**
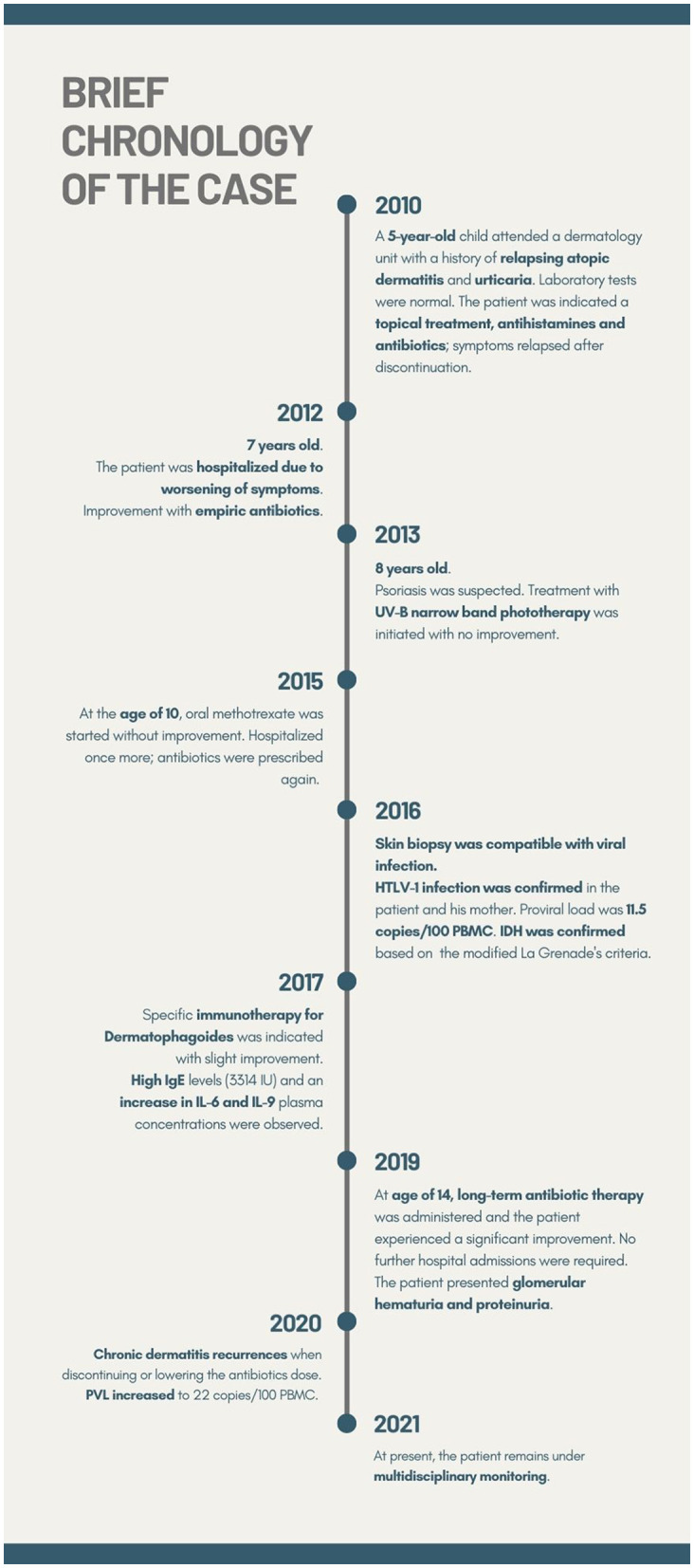
Timeline outlining the chronology for this case. HTLV-1, human T-lymphotropic virus; PBMC, peripheral blood mononuclear cells; IDH, infective dermatitis associated with HTLV-1; IU, international units; IL, interleukin; PVL, proviral load.

Currently, the severity of outbreaks is lower (with scattered skin lesions, mild eczema and papules) and the patient leads a normal social life. He started practicing physical activities and playing soccer. It should be noted that the patient's dyslipidemia improved with changes in lifestyle and diet. At present, the patient remains under multidisciplinary monitoring. New blood and urine controls, PVL determination, and a renal biopsy have been scheduled. A brief outline of the chronology of this case is provided in Figure 2.

## Discussion

We present the first pediatric case of infective dermatitis associated with HTLV-1 in Argentina. A large number of articles on epidemiology and immuno-virology associated with ATLL and HAM/TSP have been published in the scientific literature; however, there are few reports about IDH. Besides, only 5% of HTLV-1 infected individuals develop associated diseases, suggesting host genetic risk factors, such as human leukocyte antigen (HLA) haplotypes (10–12). Different therapeutic strategies are indicated but no specific treatments have been developed yet (13, 14).

Mother to child transmission through breastfeeding has been associated with IDH in childhood and ATLL in adults (15). There is evidence that HTLV-1 individuals are susceptible to various opportunistic infections and inflammatory or pulmonary diseases. Moreover, IDH could be a risk factor for progression toward both HAM/TSP and ATLL (16). Skin lesions are the first manifestations of ATLL in 50% of the patients, as has been reported in a study carried out in France (17). The diagnostic challenge resides in the fact that IDH mimics common eczemas. In Brazil, 37.5% of patients with ATLL affecting the skin were reported to have a history of severe eczema in childhood with characteristics similar to those of IDH (18). On the other hand, HTLV-1 is not commonly suspected in non-endemic areas and IDH is not well-known. In this case, an atopic dermatitis had been considered as the first diagnosis.

This patient was breastfed until the age of five by his mother who was unawarely living with HTLV-1. This factor represents a great risk for both diseases, IDH and ATLL. High PVL values have been associated with an increased risk of progression to HAM/TSP (19–21). However, in children with IDH, it has been documented that high PVL is not indicative of progression to HAM/TSP and that PVL values do not decrease after IDH remission. Conversely, higher PVL values can be associated with remission from this disease (5). In our patient, IDH symptoms improved by the age of fifteen, but in contrast with the general idea that IDH consistently improves by puberty, the patient only showed partial remission (5). However, an increase in the PVL values in time was observed. This data suggest that the disease progressed favorably, and could even be heading toward remission. Nonetheless, and even when no HTLV-1 disease intrafamilial antecedents have been observed in this case, progression to HAM/TSP cannot be discarded, and therefore monitoring is recommended.

Regarding the renal affection, it has been recently hypothesized that the chronic inflammatory state associated with HTLV-1 infection may be involved in its development (22). However, this has been reported specifically among the adult Australian aboriginal population with very high PVL. On the other hand, a case of IDH associated with HAM/TSP was reported in a 22-year-old boy who died of kidney failure (4). In our case, kidney affection not related to HTLV-1 infection has been documented in the family. The patient is now being followed-up by the nephrology unit at the hospital, and a renal biopsy is being considered.

HTLV-1 preferentially infects CD4^+^CD25^+^CCR4^+^ T cells. Tax and HTLV-1 bZIP factor (HBZ) are viral products associated with oncogenic transformation. They regulate transcription of viral proteins, host factors and cellular signaling pathways, resulting in the development of HTLV-1 associated diseases. HTLV-1 infection induces cytokine and chemokine deregulation. Increased concentrations of IL-1β, IL-6, granulocyte-macrophage colony-stimulating factor (GM-CSF), CXCL10 and IFN-γ have been reported in HAM/TSP cerebrospinal fluid, and recently, an increased concentrations of IFN-γ in plasma have been reported to be a biomarker of progression to the intermediate syndrome status and HAM/TSP (23). In this patient, IL-6 and IL-9 were elevated in plasma, but not IFN-γ, which suggests that risk of HAM/TSP is not yet present.

In relation to IL-6, it is known to be involved in overall skin immunity and resident microbial populations, and it is secreted as a response to bacterial infections (24). IL-9, secreted by Th9 skin-tropic *T* cells, acts as a potential mediator of cutaneous pathology. The majority of the reports published on these cells concern allergic skin disorders (atopic dermatitis) but Th9 cells have also been involved in inflammatory and neoplastic disorders of the skin such as psoriasis and cutaneous *T* cell lymphoma (25). IDH cases may be mistaken for many common dermatological conditions, such as atopic dermatitis and contact dermatitis. Besides, epidermal hyperplasia may mimic psoriasis, as occurred in this case, and delay the appropriate diagnosis. In contrast, plasma cells in the dermal infiltrates could contribute to prevent confusion of IDH with psoriasis (26). In this case, the typical lesions in the antecubital and popliteal fossae of atopic dermatitis were not present. In contrast, the lesions were present in three or more areas of the body (scalp, retroauricular areas, neck, axillae, groin, trunk) with recurrent relapses, and HTLV-1 infection was confirmed, which fulfilled the principal criteria for a diagnosis of IDH (4).

It has been demonstrated that the expression of both IL-6 and IL-9 is activated by the HTLV-1 oncoprotein Tax *via* a nuclear factor kappa-light-chain-enhancer of activated B cells (NF-κB) motif in its proximal promoter (27) and a role for IL-9 and its receptor in ATLL by a paracrine mechanism has been reported (28).

On the other hand, as suggested by McGill et al. (7), increased IgE levels were associated with bacterial superinfection in children with atopic dermatitis (29), as well as single nucleotide polymorphisms (SNPs) in genes associated with maintaining the integrity of the skin barrier, as is filaggrin (30). Since IgE levels are commonly found in patients with IDH (3), as is this case, it has been hypothesized that there is a link between these levels and the frequent *Staphylococcus aureus* infection in patients with IDH. Omalizumab, a human monoclonal IgG anti-IgE antibody, is an alternative treatment for chronic urticaria (31, 32). This drug is currently not indicated for IDH, but it would be interesting to analyze whether it could also be a treatment strategy for this pathology.

Considering this data and the fact that HTLV-1 may induce systemic effects, the patient will be followed-up in time with the objective to detect associated pathologies from an early stage, in the case any of them is developed. Monitoring includes blood testing, clinical neurological exam, and measurements of PVL and IFN-γ levels. Besides, circulating atypical lymphocytes (“flower” cells) are pathognomonic of ATLL, so peripheral blood smear is also relevant (33).

HTLV-1 is a neglected virus, recently recognized by the World Health Organization in the last report published in March 2021 (34). Antibody HTLV-1 testing in blood banks is mandatory in several countries, among which is Argentina, but this retrovirus has not been included yet in National Health programs to implement mother-to-child and sexual transmission surveillance, nor testing in human milk banks. Lack of diagnostic guidelines results in scarce knowledge to address HTLV-1 infection by the medical community leading to late diagnosis and possibly, sub-diagnosis of these pathologies. Taking this into consideration, it is possible that more cases of IDH exist in Argentina, particularly in the northwestern endemic region, as has been reported in other HTLV-1 endemic areas, such as Bahia, in Brazil (4). Considering that there are distinct endemic areas for HTLV-1 worldwide and the frequent migration to/from non-endemic regions, the need for international guidelines and surveillance of HTLV-1 becomes crucial.

An accurate diagnosis of IDH in infants is relevant to indicate the appropriate treatment and to monitor the possible development of other systemic related diseases (35). It is advisable to suspect HTLV-1 infection in cases that exhibit chronic dermatosis and intermittent response to antibiotic treatment. It is recommended that a multidisciplinary team carry out long-term follow-up of these children. An improved understanding of the immunopathological mechanisms of this disease to enable the development of specific treatments is vital.

## Data Availability Statement

The raw data supporting the conclusions of this article will be made available by the authors, without undue reservation.

## Ethics Statement

Ethical review and approval was not required for the study on human participants in accordance with the local legislation and institutional requirements. Written informed consent to participate in this study was provided by the participants' legal guardian/next of kin. Written informed consent was obtained from the minor(s)' legal guardian/next of kin for the publication of any potentially identifiable images or data included in this article.

## Author Contributions

PB: data collection and analysis, writing, edition, and revision of the manuscript. ND: data analysis and revision of manuscript. LA, II, LP, ML, and MBe: data collection and revision of the manuscript. CB and MBi: conceptualization, supervision, writing, edition, and revision of the manuscript. All authors read and approved the final manuscript.

## Funding

This work was supported by the Argentinean Ministry of Science, Technology and Innovation (grant number PICT 2019-1633).

## Conflict of Interest

The authors declare that the research was conducted in the absence of any commercial or financial relationships that could be construed as a potential conflict of interest.

## Publisher's Note

All claims expressed in this article are solely those of the authors and do not necessarily represent those of their affiliated organizations, or those of the publisher, the editors and the reviewers. Any product that may be evaluated in this article, or claim that may be made by its manufacturer, is not guaranteed or endorsed by the publisher.

## Abbreviations

HTLV-1: human T-lymphotropic virus
ATLL: adult T-cell leukemia/lymphoma
HAM/TSP: HTLV-1 associated myelopathy/tropical spastic paraparesis
IDH: infective dermatitis associated to HTLV-1
PVL: proviral load
TNF-α: tumor necrosis factor alpha
IFN-ɤ: interferon gamma
T/S: trimethoprim/sulfamethoxazole
NB-UVB: narrow band ultraviolet B
ELISA: enzyme-linked immunoassay
n-PCR: nested polymerase chain reaction
PBMC: peripheral blood mononuclear cells
qPCR: quantitative polymerase chain reaction
HIV: human immunodeficiency virus
MRI: magnetic resonance imaging
RAST: radio allergo sorbent test
IL: interleukin
HLA: human leukocyte antigen
HBZ: HTLV-1 bZIP factor
GM-CSF: granulocyte-macrophage colony-stimulating factor
NF-kB: nuclear factor kappa-light-chain-enhancer of activated B cells
SNPs: single nucleotide polymorphisms.
